# Online Tiered Screening for Mental Health Problems Among Refugees in Sweden: Validation Study

**DOI:** 10.2196/82763

**Published:** 2026-01-29

**Authors:** Jennifer Meurling, Elisabet Rondung, Youstina Demetry, Anahita Geranmayeh, Anna Leiler, Gerhard Andersson, Anna Bjärtå

**Affiliations:** 1 Department of Education, Psychology and Social Work Mid Sweden University Östersund Sweden; 2 Centre for Psychiatry Research Department of Clinical Neuroscience Karolinska Institute Stockholm Sweden; 3 Department of Behavioral Sciences and Learning Linköping University Linköping Sweden

**Keywords:** asylum-seekers, diagnostic test accuracy, digital mental health, eHealth, mHealth, online assessment, refugees, tiered screening, validation

## Abstract

**Background:**

Refugees and asylum-seekers commonly experience numerous adverse and traumatic events and are therefore at increased risk of mental health problems. Despite the high need for mental health interventions, services tend to be underused by refugees and asylum-seekers, and various barriers compromise access. Digital, efficient screening, adapted for these groups, could facilitate initial assessment and increase accessibility to mental health services. We developed an internet-based tiered screening procedure (i-TAP) aiming to identify clinically relevant symptoms of major depressive disorder (MDD), anxiety disorder, posttraumatic stress disorder, and insomnia disorder among individuals with a refugee background. The i-TAP is an adaptive procedure with 3 tiers aiming to identify general mental distress in Tier 1, differentiate between symptoms in Tier 2, and assess the severity of symptoms in Tier 3. Each tier additionally functions as a gateway to further assessment, as a negative outcome terminates the procedure.

**Objective:**

The purpose of this study was to evaluate the diagnostic test accuracy of the i-TAP, using structured clinical assessments as the reference standard.

**Methods:**

In this prospective study, 70 adult participants with a refugee background, literate in Arabic, Dari, Farsi, or Swedish, and residing in Sweden, completed the i-TAP on tablets and participated in a subsequent structured diagnostic interview.

**Results:**

It has been shown that the i-TAP could identify 91.7% (33/36) of individuals assessed with any psychiatric disorder, and correctly identified 82.1% of all positive cases of MDD, anxiety disorder, posttraumatic stress disorder, and insomnia disorder, with few false negative assessments. Overall test accuracy of the i-TAP ranged between 77.1% and 84.3%, depending on disorder. The tiered design could reduce item burden while maintaining accuracy. A vast majority of participants rated the user experience as positive. In this sample, 36/70 (51.4%) individuals were assessed with one or more psychiatric disorders and comorbidity was high.

**Conclusions:**

The i-TAP may be a valid, efficient, and feasible screening tool for the identification of common psychiatric disorders among individuals with a refugee background in Sweden. The i-TAP could be implemented as a first screener in various settings, including online and in-person clinical practices. The digital, adaptive, multilingual format could facilitate early assessment and increase the availability of mental health services for refugees and asylum-seekers.

## Introduction

### Background

According to the United Nations High Commissioner for Refugees (UNHCR), there were an estimated 123.2 million forcibly displaced people in the world by the end of 2024 [1]. This number is expected to rise due to ongoing conflicts and wars globally. UNHCR reserves the term refugees for forcibly displaced people who, due to violence, war, conflict, or persecution, have crossed international borders to seek safety in another country, and the term asylum-seeker for people who have applied for refugee status and are awaiting a decision [2]. In Sweden, asylum-seekers have come mainly from Syria, Afghanistan, Iraq, Eritrea, Somalia, and Iran in the years 2015-2025 [3]. Since 2022, refugees from Ukraine have been treated under the European Union Temporary Protection Directive.

In addition to having fled their homes, many refugees and asylum-seekers have endured numerous stressful and potentially traumatic events, increasing the risk of psychological distress and various mental disorders [4-6]. When arriving in their host countries, refugees are further exposed to postmigration stressors, such as language barriers, discrimination, long asylum processes, and economic as well as social challenges, all of which are associated with poor mental health [4,6,7].

Posttraumatic stress disorder (PTSD), major depressive disorder (MDD), and anxiety disorders are highly prevalent among refugees [8,9], with even higher rates observed among asylum-seekers [10,11]. Symptoms persist over time [9,12] and often co-occur [11]. Furthermore, insomnia disorder is highly prevalent [13,14] and various sleep disturbances are reported as problematic [15]. Although prevalence rates vary between studies of refugee mental health, consensus in the field establishes significantly elevated levels of psychiatric symptoms and disorders among refugee groups, including asylum-seekers, compared to the general population [8,9,16]. Despite the high prevalence of mental health issues, mental health services tend to be underused [17,18]. This discrepancy, or treatment gap, can be understood through barriers frequently reported by both help-seeking refugees and asylum-seekers, and service providers. These barriers include language obstacles and the need to use interpreters, difficulties navigating the health care system, and fear of stigma [17,19], along with practical issues and financial concerns [20], all of which affect accessibility to relevant services.

Given that the mental health of individuals who have fled their homelands is affected by adversity experienced before, during, and after the flight [6], the increased risk of mental health problems persists over time, also long after resettlement [9,12]. This underscores the importance of identifying those in need of interventions, regardless of the duration of displacement. In a recent review [21], systematic mental health screening with at least 2 screening occasions is recommended for recently resettled refugees and asylum-seekers. Early and repeated mental health screening could increase access to and provision of treatment, ultimately reducing unnecessary suffering from untreated symptoms. This emphasizes the need for validated, efficient, and feasible methods to identify common mental health problems among individuals who have fled their homelands.

Offering internet-based mental health screening could increase accessibility of mental health services by addressing several of the aforementioned barriers. For instance, internet-based and smartphone-assisted options provide flexibility and privacy, can be time-saving, and can easily be offered in multiple languages [22,23]. Although the literature on this topic is limited, digital mental health screening tools have previously been used among refugees and asylum-seekers [24], with evidence supporting their acceptability, feasibility, and validity for these populations [25,26]. Furthermore, a digital format enables the use of adaptive hierarchical screening models, which allow simultaneous screening of multiple psychiatric symptoms and can reduce the overall respondent burden while maintaining high accuracy [27,28].

Recognizing the potential technical as well as social advantages of online mental health screening for refugees and asylum-seekers, we set out to design and evaluate an internet-based tiered assessment procedure (i-TAP). The i-TAP is tailored for refugee groups residing in Sweden, and specifically designed to identify clinically relevant symptoms of depression, anxiety, PTSD, and insomnia.

### The i-TAP

**Figure 1:**
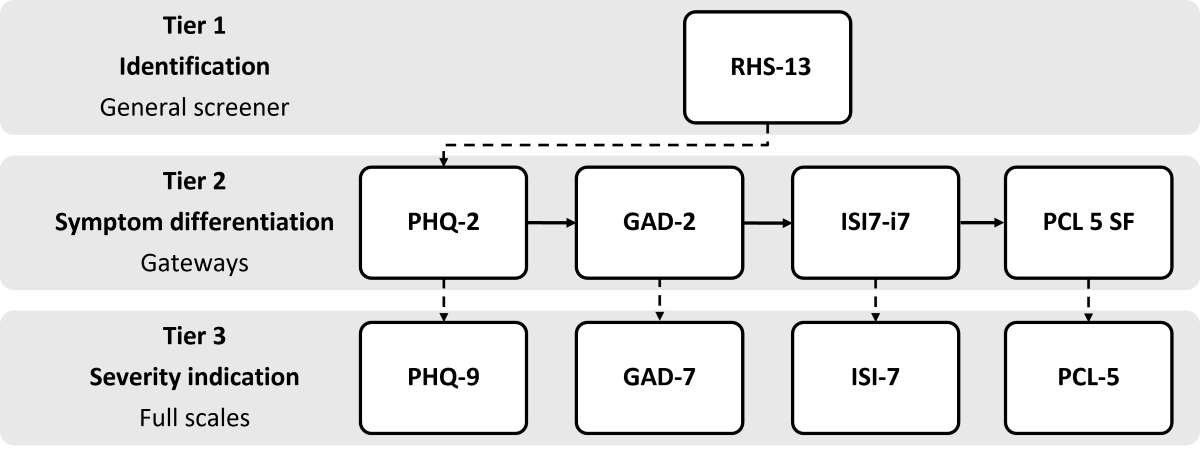
Illustration of the internet-based tiered screening procedure, i-TAP, with the symptom scales used in each tier. GAD-2: Generalized Anxiety Disorder-2; GAD-7: Generalized Anxiety Disorder-7; Insomnia Severity Index-7, item 7; ISI-7: Insomnia Severity Index-7; PCL 5: PTSD (posttraumatic stress disorder) Checklist for DSM-5 (Diagnostic and Statistical Manual of Mental Disorders [Fifth Edition]); PCL-5 SF: PTSD (posttraumatic stress disorder) Checklist for DSM-5 (Diagnostic and Statistical Manual of Mental Disorders [Fifth Edition]) Short Form; PHQ-2: Patient Health Questionnaire-2; PHQ-9: Patient Health Questionnaire-9; RHS-13: Refugee Health Screener-13.

In a previous study by Meurling et al [28], the instruments and the procedure constituting the i-TAP were evaluated, yielding a model designed for optimal psychometric performance, along with promising results regarding the efficiency, including a reduced item burden and accuracy of the procedure. The i-TAP is a 3-tiered screening procedure that adapts to the respondent’s answers in each tier. The first tier is highly sensitive, aiming to identify general mental distress while preventing further assessment of individuals without psychiatric symptoms. Thus, a negative outcome in Tier 1 terminates the procedure, while a positive outcome forwards the respondent to Tier 2. The second tier comprises brief symptom scales and differentiates between symptoms of depression, anxiety, PTSD, and insomnia. It also serves a gateway function, forwarding individuals with a positive outcome on one or more scales to further assessment in Tier 3, while terminating the procedure for respondents with a negative outcome on all Tier 2 scales. In the third tier, the full scales corresponding to the outcome of Tier 2 are presented to the respondent, with the purpose of indicating severity and identifying clinically relevant symptoms of depression, anxiety, PTSD, and insomnia. The items already answered in Tier 2 have been removed from each full scale in Tier 3. The tiered design of the i-TAP with the included instruments is depicted in Figure 1. In both the initial study [28] and this one, the i-TAP was provided in Arabic, Dari, Farsi, English, and Swedish, based on the largest groups of refugees and asylum-seekers in Sweden at the time the study commenced [29].

In Meurling et al [28], the Tier 1 and Tier 2 scales were evaluated using the Tier 3 scales as the reference standard. We also applied receiver operating characteristic analysis to identify gateway cutoffs for optimal performance. This resulted in an accurate screening tool (total precision 86%) that could identify most moderate symptoms of PTSD, depression, anxiety, and/or insomnia (96.2%). These results and the fact that only the first 2 tiers of the model were tested prompted further evaluation of the full 3-tiered model. The developmental process of the i-TAP is described in further detail in Meurling et al [28].

### Aim

The aim of this study is to test the performance of the full i-TAP. Specifically, we seek to evaluate the criterion validity by assessing diagnostic test accuracy of the full tiered model, using structured clinical assessments of psychiatric disorders as the reference standard. Our objective is to determine the extent to which the i-TAP is able to identify MDD, anxiety disorder, PTSD, and insomnia disorder among individuals with a refugee background, residing in Sweden. We want to emphasize that the heightened risk for these disorders follows experiences of flight and resettlement. The target group for the i-TAP is therefore focused on the refugee experience, irrespective of current legal status. Accordingly, we henceforward use the term individuals with a refugee background to describe our study population.

## Methods

### Study Context and Design

This study and the development of the i-TAP are part of the SAHA project, a collaboration between Mid Sweden University, Linköping University, and Karolinska Institute, with the overarching aim of developing digital mental health solutions, including tailored interventions, for individuals with a refugee background. We conducted this prospective cross-sectional validation study with individuals with a refugee background residing in Sweden, collecting data from June to October 2022.

### Recruitment and Participants

Participants were recruited through convenience sampling in various nonclinical settings (adult education for immigrants, language cafés, nongovernmental organizations, and at an asylum housing facility). Recruitment took place on site with the help of local personnel and interpreters. After being informed about the study orally, interested individuals could approach immediately or later via email or phone. The inclusion criteria were refugee background, aged 18 years or older, literacy in Arabic, Dari, Farsi, Swedish, or English, and currently living in Sweden. Being a refugee or having a refugee background was herein specified as having fled to Sweden due to war, conflict, persecution, threat, or similar reasons, and was self-defined by the participants. This criterion was carefully explained during recruitment and verified before starting each study procedure, to ensure inclusion of participants with a refugee experience. Individuals were thus included regardless of residence status, and we have not differentiated analyses based on that.

### Data Collection and Material

#### Overview

Data collection was carried out by 11 persons trained in clinical psychology and with practical experience of psychiatric assessment, herein referred to as psychologists. The assessments were conducted in each respondent’s preferred language. Five of the psychologists were bilingual and 6 psychologists used authorized interpreters via telephone to conduct the assessments. To facilitate translation, the assessment material was sent to interpreters in advance. All psychologists lived in Sweden and were fluent in Swedish. To ensure methodological equivalence, all psychologists received training in the full procedure prior to data collection.

Data collection included digital screening with the i-TAP, followed by a structured clinical interview. The whole procedure was carried out by a psychologist on a single occasion in a private space adjacent to each recruitment site. Each occasion lasted a maximum of 2 hours and was preceded by oral and written informed consent from the participant.

#### Digital Screening

Participants completed the i-TAP independently, online via tablets. For this study, we also included background questions and feedback questions regarding the experience of completing the i-TAP. The psychologists (and telephone interpreters) were available to answer any questions but were instructed not to sit next to or help the participants fill in the survey. The results of the i-TAP were blinded to the psychologists.

#### Structured Diagnostic Interview

The structured diagnostic interview targeted the same disorders as the i-TAP, namely insomnia, PTSD, MDD, and anxiety disorders (panic disorder, agoraphobia, social anxiety disorder, and generalized anxiety disorder [GAD]). Before terminating the clinical interview, the psychologist invited the participant to add any information or raise other topics of their choice. To conclude the procedure, the psychologist summarized the diagnostic interview and gave participants feedback and recommendations based on their assessment. All participants received a brief written summary of the clinical assessment, recommendations (if applicable), a folder with contact information to available local and national health care services, and a SEK 99 (US $9.7) gift card to a supermarket. We offered to guide participants in help-seeking, for example, by showing websites, finding opening hours and contact information, and exploring maps. If an immediate risk was identified, this was handled according to a predefined safety protocol described below. To ensure confidentiality, data were pseudonymized. In cases where the procedure was terminated before completion, participants were also offered the information folder and gift card.

Each clinical interview was discussed at team meetings, referred to as clinical conferences, before determining the final clinical assessment of each participant. The psychologists were still blinded to the results of the i-TAP during these discussions. The clinical conferences promoted both discussion and unanimity in the final assessments.

### Index Test

The i-TAP builds on 56 items from 5 validated scales, described in detail below. The symptom scales were selected based on psychometric properties, cross-cultural validity or previous use in refugee populations (or a combination thereof). All scales have previously been evaluated by us for the current population [28,30]. For a detailed description of the scales, translation, and psychometric properties, please see our previous publication from the SAHA project [28]. All scales use Likert response alternatives. For the analyses in this study, responses were dichotomized using cutoffs on each scale. Considering that i-TAP aims for high diagnostic sensitivity yet being an efficient screening procedure, we have applied the sensitivity model proposed in our first study [28] for this study. That is, we have applied cutoffs to identify as many true positives as possible without compromising accuracy.

The following instruments and cutoff values for moderate symptoms were used: for identification of general psychological distress in Tier 1, we used the Refugee Health Screener-13 (RHS-13) [31,32] with cutoff ≥11. For symptoms of MDD, we used the Patient Health Questionnaire-2 (PHQ-2) [33], with cutoff ≥2 in Tier 2 and the Patient Health Questionnaire-9 (PHQ-9) [34] with cutoff ≥10 in Tier 3. Symptoms of anxiety disorders (including panic disorder, social anxiety disorder, and GAD), were measured using the Generalized Anxiety Disorder-2 (GAD-2) [35], with cutoff ≥2 in Tier 2 and the Generalized Anxiety Disorder-7 (GAD-7) [36,37] with cutoff ≥10 in Tier 3. Selection of the anxiety scales was based on very good psychometric properties when screening for GAD, panic disorder, and social anxiety disorder [37], which enables the use of 1 instead of 3 separate anxiety scales, reducing the item burden for the respondents. This aligns with the aim of the i-TAP to efficiently detect clinically relevant symptoms of a range of anxiety disorders. Symptoms of PTSD were measured with the PTSD Checklist for *DSM-5* (*Diagnostic and Statistical Manual of Mental Disorders* [*Fifth Edition*]) Short Form (PCL-5 SF) [38], using cutoff ≥5 in Tier 2 and the PTSD Checklist for *DSM-5* (PCL-5) [39] using cutoff ≥32 in Tier 3. To identify symptoms of insomnia, we used the Insomnia Severity Index-7 (ISI-7) [40], item 7 with cutoff ≥2 in Tier 2 and the full scale in Tier 3. For ISI-7, we previously used a conservative cutoff for detecting moderate to severe symptoms of insomnia (≥14). However, 2 more sensitive cutoffs have been proposed, ≥8 and ≥11 [41,42]. We evaluated the performance of all 3 cutoffs and concluded that cutoff 11 was best suited for the purpose of the i-TAP. Please see Table S1 in Multimedia Appendix 1 for detailed results of this analysis. All Tier 2 instruments are composed of items from the Tier 3 instruments. To avoid posing the same questions twice, these items were not repeated in Tier 3. Instead, we included the item responses from Tier 2 in the analyses of the full scales in Tier 3.

To assess user experience of the i-TAP, we formulated 3 items about answering questions about mental health online. The first assessed if participants had a negative or positive attitude (1-10 with 1=very negative and 10=very positive), the second assessed if the i-TAP was difficult or easy to use (1-10 with 1=very difficult and 10=very easy), and the third assessed if the questions in the i-TAP were difficult or easy to understand (1-5 with 1=very difficult, 2=difficult, 3=neither difficult nor easy, 4=easy, and 5=very easy to understand).

### Reference Standard

The reference standard was a clinical diagnosis of MDD, anxiety (panic disorder, agoraphobia, social anxiety disorder, and GAD), PTSD, or insomnia according to the clinical assessment. The assessment of clinical diagnoses relied on a structured diagnostic interview, including sections investigating insomnia disorder, trauma experience, PTSD, MDD, panic disorder, agoraphobia, social anxiety disorder, and GAD, in the presented order. Structured diagnostic interviews are generally accepted as the gold, or reference standard in clinical research [43].

The principal instrument of the structured diagnostic interview was the Mini International Neuropsychiatric Interview 7.0.0 (M.I.N.I.) [44]. M.I.N.I. is a short, structured psychiatric interview based on *DSM-5* (*Diagnostic and Statistical Manual of Mental Disorders, Fifth Edition*) and *ICD-10* (*International Statistical Classification of Diseases, Tenth Revision*) diagnostic criteria and has demonstrated excellent test and interrater reliability [45]. It was originally designed for epidemiological and multicenter studies and is now used extensively in both research and clinical settings, as well as in global mental health settings [46] and with refugee populations [16,47]. The M.I.N.I. is designed to be sensitive [44] and has repeatedly been proven so in comparison with expert clinical diagnoses [43,46]. In this study, we used the diagnostic sections for PTSD, major depressive episode, panic disorder, agoraphobia, social anxiety disorder, and GAD. No official validated translations of the M.I.N.I. were available. For Arabic, we used a previously evaluated translation [46], and the bilingual psychologists made translations to Dari and Farsi. As insomnia is not included in the M.I.N.I., we designed a module for this specific diagnosis comprising 6 questions responding to the diagnostic criteria in *DSM-5* [48], matching the format in the M.I.N.I. to facilitate administration. We formulated brief questions to simplify interpreter-assisted assessments. The complete insomnia assessment is depicted in Multimedia Appendix 1. Furthermore, we adapted the M.I.N.I. to the target population by replacing the suicidality module and the criterion A assessment for PTSD (see details below).

To investigate potentially traumatic events (PTEs), we used the Refugee Trauma History Checklist (RTHC), a measure developed for adult refugees and previously validated in a sample of Syrian refugees in Sweden [49]. The RTHC is designed to assess the most common refugee-related PTEs, before, during, and after flight, in a nonintrusive way. The checklist is composed of 2 questions, where the respondent is asked if they have experienced any of 7 or other PTEs before and after they left their home (war at close quarters, forced separation from family or close friends, loss or disappearance of family members or loved ones, physical violence or assault, witnessing physical violence or assault, torture, sexual violence, and other frightening situation where you felt your life was in danger). Participants were not asked to elaborate or describe reported events further. We used the RTHC as a replacement for item H1 in the M.I.N.I., which corresponds to the criterion A assessment for PTSD in *DSM-5*; thus, a positive RTHC outcome regarding any traumatic life event was followed by continued PTSD assessment with the M.I.N.I. This adaptation was made against previous research pointing toward the importance of broadening the trauma criterion and description in *DSM-5* for individuals with a nonwestern background [50].

To assess suicidality, we used the Columbia Suicide Severity Rating Scale Screening Version (C-SSRS Screen) [51,52] as a structured interview. The C-SSRS Screen has 6 items with a similar structure to the M.I.N.I., is validated as an initial step to identify individuals with short-term suicide risk [51], and has previously been used in refugee populations [53]. A positive outcome was handled according to a predefined safety protocol, including immediate consultation with relevant health services, together with participants, for thorough suicide risk evaluation. If suggested, we offered to accompany the participant to the care facility. We included suicidality as part of the clinical assessment to identify any individuals in need of immediate clinical attention; however, suicidality was not part of this validation study. Against this, and to further adapt the structured assessment to the target group, we decided to replace the extensive and sometimes linguistically complicated suicidality module in the M.I.N.I. with a shorter, more comprehensible screener.

### Data Treatment and Analysis

Sample size requirements were estimated based on expected prevalence and desired sensitivity. Based on previous studies of refugee mental health [8,9,16], we expected a high prevalence (>30%) of moderate symptoms of each disorder and an even higher prevalence based on total outcomes from the i-TAP. According to Bujang and Adnan [54], a sample size of 67 is adequate to determine accuracy (80% power and significance level <.05) based on a minimum prevalence of 30%, and the aim of 80% sensitivity we set for the i-TAP.

The structured diagnostic interview was assessed and summarized as positive (“diagnosis”) or negative (“no diagnosis”) for each diagnosis. A diagnosis is herein defined as fulfilling diagnostic criteria for a psychiatric disorder according to clinical assessment with M.I.N.I. and the structured clinical interview for insomnia. Panic disorder, agoraphobia, social anxiety disorder, and GAD in M.I.N.I. were collapsed into one variable, anxiety disorder*,* for the analysis.

Descriptive measures were summarized as frequencies and proportions or means and SDs depending on variable type. Internal consistency was calculated for the full scales included in the i-TAP using Cronbach alpha. Intercorrelations were assessed using Pearson *r*.

We tested the criterion validity of the i-TAP by comparing the outcome of the complete i-TAP procedure for each symptom category (depression, anxiety, PTSD, and insomnia) with the corresponding clinical diagnoses according to the reference standard. We used Cohen κ as a statistically adjusted measure of concordance between 2 measures [55]. Interpretation of κ values was guided by Landis and Koch [56] suggested labels: <0 no agreement, 0-0.20 slight, 0.21-0.40 fair, 0.41-0.60 moderate, 0.61-0.80 substantial, and 0.81-1.0 perfect agreement. Diagnostic test accuracy, including sensitivity, specificity, positive and negative predictive values [57], and the number of false negatives and false positives, was calculated for each tier and symptom category, using clinical diagnosis from the structured clinical interview as the reference standard.

All individuals had complete data on all scales and were included in the analysis. Analyses were performed using IBM SPSS (version 29).

### Ethical Considerations

The study protocol has been approved by the Swedish Ethical Review Authority (2020-00214). This study was planned and performed in accordance with the ethical standards of the 1975 Helsinki declaration and its amendments (World Medical Association, 2001). Written informed consent was obtained from all participants, in their preferred language, prior to study participation. Participants were carefully informed that participation was voluntary and data confidential, that they could terminate their participation at any time, and that participation could not affect the asylum process. To protect the privacy of participants, data are presented at the group level, and potentially identifying details regarding participants or data collection have been omitted.

## Results

### Sample

In total, 85 individuals registered interest to participate in the study; however, 6 were lost due to nonattendance. Out of the 79 that initiated assessments, 7 did not complete for reasons such as low literacy, not having a refugee background, or practical circumstances. We completed assessments with 72 participants. Due to technical issues, data were incomplete for 2 respondents, rendering a final sample of 70 participants. Against the power calculation, our sample size of 70 was deemed sufficient.

### Clinical and Sociodemographic Characteristics

Out of the 70 analyzed assessments, 31 were conducted in Arabic, 14 in Dari, 15 in Farsi, and 10 in Swedish. Participants were 31 females and 39 males, between 19 and 69 years old (mean 39.1, SD 13.16), and 31.4% (22/70) lived at an asylum facility, 50% (35/70) had their own housing, and 18.6% (13/70) lived together with family or friends, see Table 1 for sociodemographic characteristics of the sample.

**Table 1:** Sample characteristics of participants (N=70).

Characteristic		Participants, n (%)
**Nationality**		
	Syria	25 (35.7)
	Afghanistan	19 (27.1)
	Iran	12 (17.1)
	Palestine	5 (7.1)
	Other (n<5)^a^	9 (12.9)
**Sex**		
	Female	31 (44.3)
	Male	39 (55.7)
**Marital status**		
	Single	17 (24.3)
	Married or partner	44 (62.9)
	Divorce or separated	6 (8.6)
	Widowed	2 (2.9)
	Other	1 (1.4)
**Time in Sweden**		
	<1 year	10 (14.3)
	1-3 years	17 (24.3)
	4-6 years	19 (27.1)
	7-10 years	24 (34.3)
**Education**		
	University Master of Arts	10 (14.3)
	University Bachelor of Arts	10 (14.3)
	High school	25 (35.7)
	Primary school	19 (27.1)
	Vocational qualification	3 (4.3)
	Other	3 (4.3)
**Residence permit**		
	No residence permit	19 (27.1)
	Temporary	18 (25.7)
	Permanent	33 (47.1)

^a^Iraq, Eritrea, Tajikistan, Somalia, Sudan, and Kurdistan.

Each scale in the i-TAP demonstrated excellent internal consistency, with Cronbach α of 0.93 for RHS-13, 0.92 for PHQ-9, 0.92 for GAD-7, 0.96 for PCL-5, and 0.92 for ISI-7. All scales were intercorrelated, *r* ranging from 0.70 to 0.89 (all *P*<.001). Regarding symptom assessment, the mean for RHS-13 was 21.61 (SD 13.95; 95% CI 18.29-24.92), for PHQ-9, 9.86 (SD 7.20; 95% CI 8.14-11.57), for GAD-7, 8.33 (SD 6.43; 95% CI 6.79-9.86), for PCL-5, 29.99 (SD 22.74; 95% CI 24.56-35.41), and the mean for ISI-7 was 11.64 (SD 7.96; 95% CI 9.75-13.54).

Prevalence of clinical diagnoses of MDD, anxiety disorder, PTSD, and insomnia disorder was high in the sample, see Table 2 for prevalence estimates for each disorder by the i-TAP and the clinical assessment, respectively. All participants reported experiencing at least one potentially traumatic event. According to the structured diagnostic interview, 51.4% (36/70) met diagnostic criteria for one or more disorders.

**Table 2:** Prevalence estimates assessed with the structured diagnostic interview and the i-TAP^a^, respectively (N=70)^b^.

Disorder		Clinical assessment (diagnosis), n (%)	i-TAP (moderate symptoms), n (%)
Major depressive disorder		20 (28.6)	33 (47.1)
**Anxiety disorder (any)^c^**		17 (24.3)	25 (35.7)
	Panic disorder	5 (7.1)	—^d^
	Agoraphobia	0 (0)	—
	Social anxiety disorder	3 (4.3)	—
	Generalized anxiety disorder	14 (20.0)	—
PTSD^e^		20 (28.6)	27 (38.6)
Insomnia disorder		26 (37.1)	32 (45.7)
Any^f^		36 (51.4)	41 (58.6)

^a^i-TAP: internet-based tiered screening procedure.

^b^Multiple diagnoses per individual are possible. Clinical assessment=assessment results of the structured diagnostic interview, i-TAP=individuals who scored positive in Tier 1, Tier 2, and above the cutoff for moderate symptoms in Tier 3.

^c^Positive case for any anxiety disorder.

^d^Not applicable.

^e^PTSD: posttraumatic stress disorder.

^f^Positive case for any disorder.

Comorbidity was generally high. The clinical assessment showed that 23 individuals (32.9%) fulfilled criteria for 2 or more diagnoses, of which 8 individuals met criteria for 3 diagnoses, and 8 for all 4 diagnoses. Comorbidity rates per identified disorder are depicted in Table 3. Among the 13 individuals who fulfilled criteria for one diagnosis only, insomnia was most prevalent (n=7).

**Table 3:** Comorbidity of disorders diagnosed with the structured diagnostic interview (N=70)^a^.

Diagnosis	Number of participants	Comorbid MDD^b^, n (%)	Comorbid anxiety disorder, n (%)	Comorbid PTSD^c^, n (%)	Comorbid insomnia disorder, n (%)
MDD	20	—^d^	—	—	—
Anxiety disorder	17	12 (17.1)	—	—	—
PTSD	20	14 (20.0)	12 (17.1)	—	—
Insomnia disorder	26	15 (21.4)	13 (18.6)	13 (18.6)	—

^a^Percentages are calculated based on the total sample (N=70).

^b^MDD: major depressive disorder.

^c^PTSD: posttraumatic stress disorder.

^d^Not applicable.

### Concordance of the i-TAP and the Structured Diagnostic Interview

The i-TAP identified 33 of the 36 (91.7%) individuals with a clinical diagnosis according to the structured diagnostic interview. The i-TAP correctly identified 19/20 cases of MDD, 13/17 cases of anxiety, 16/20 cases of PTSD, and 21/27 cases of insomnia disorder (ie, true positives) in the current sample. κ values indicated moderate concordance between the i-TAP and the clinical assessment for the specific disorders, and diagnostic test accuracy was between 77.1% and 78.6% for all 4 diagnoses. Regarding overall concordance, accuracy was 84.3% for the identification of any disorder and the overall concordance was substantial, see Table 4 for detailed results.

**Table 4:** Concordance of the i-TAP^a^ and the clinical assessment, calculated for each i-TAP disorder with clinical diagnosis according to the structured diagnostic interview as the reference standard.

i-TAP diagnosis	n_pos_^b^	Cohen κ	Sensitivity, % (95% CI)	Specificity, % (95% CI)	PPV^c^, % (95% CI)	NPV^d^, % (95% CI)	Accuracy, % (95% CI)
Depression	33	.56	95.0 (85.5-100)	72.0 (59.6- 84.5)	57.6 (40.7-74.5)	97.3 (92.0- 100)	78.6 (68.9- 88.2)
Anxiety	25	.46	76.5 (56.3- 96,7)	77.4 (66.1- 88.7)	52.0 (32.4- 71.6)	91.1 (82.8- 99.4)	77.1 (67.3- 86.9)
PTSD^e^	27	.53	80.0 (62.5-97.5)	78.0 (66.5- 89.5)	59.3 (40.8- 77.8)	90.7 (82.0- 99.3)	78.6 (68.9- 88.2)
Insomnia	32	.53	80.8 (64.6- 95.4)	75.0 (62.2- 87.8)	65.6 (49.1- 82.1)	86.8 (76.0- 97.6)	77.1 (67.3- 86.94)
Any^f^	41	.68	91.7 (82.69- 100)	76.5 (62.25- 90.75)	80.5 (68.4- 92.6)	89.7 (71.1- 97.5)	84.3 (75.8- 92.8)

^a^i-TAP: internet-based tiered screening procedure.

^b^n_pos_: number of positive cases for each diagnosis.

^c^PPV: positive predictive value.

^d^NPV: negative predictive value.

^e^PTSD: posttraumatic stress disorder.

^f^Positive case for any diagnosis.

### Precision of the i-TAP

To investigate to what level the i-TAP was able to correctly assess the prevalence of disorders on an individual level, we have calculated the number of correct assessments (ie, true positives and true negatives) performed by the i-TAP over all 4 disorders and within each individual. That is, each participant could have 0 to 4 correct assessments of diagnosis or no diagnosis.

The i-TAP could correctly assess 54.3% (n=38) of all participants. These individuals were thus correctly positively or negatively assessed on all 4 disorders. Fourteen (20.0%) individuals were correctly assessed on 3 of the 4 disorders, of which 11 (78.6%) of the failed assessments were false positives. The remaining 18 participants had 2 or less correctly identified assessments. Equally for these individuals, a vast majority of the failed assessments were false positives (77.1%). A total of 8 individuals were assessed as false positives by the i-TAP without any diagnosis.

Regarding false negative assessments, a total of 11 individuals were affected as the i-TAP failed to identify 1 case of MDD, 4 cases of GAD, 4 cases of PTSD, and 5 cases of insomnia disorder. However, only 3 individuals with a diagnosis according to the reference standard were entirely missed, as the other 8 false negative cases were identified as positive on another diagnosis.

### A Tiered Model

**Figure 2:**
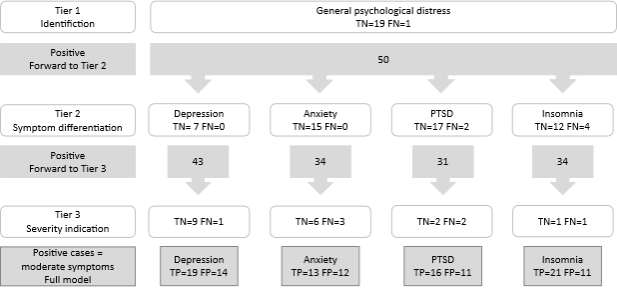
The culling effects of the hierarchical design of the internet-based tiered screening procedure (i-TAP). FN: false negatives (incorrectly excluded cases); FP: false positives (incorrectly identified cases); PTSD: posttraumatic stress disorder; TN: true negatives (correctly excluded cases); TP: true positives (correctly identified cases).

To calculate the tiered model, we used all data (all respondents answered all scales) to simulate the effects of the tiers in the i-TAP. In Tier 1, identification of symptoms, 50 individuals screened positive, indicating psychological distress in 71.4% (50/70) of the participants. Tier 1 showed very high sensitivity (97.2%), low specificity (55.9%), and correctly excluded 19 individuals (ie, true negatives) from further assessment; however, 1 individual with clinical anxiety diagnosis was falsely excluded (ie, a false negative). Tier 2, differentiation between symptoms, also exhibited high sensitivity (range 78.6%-100%) and specificity ranging between 54.0% and 74.0% for the 4 disorders. See Figure 2 for an illustration of the culling effects of the hierarchical design. Detailed results of the psychometric evaluation of each tier and disorder are depicted in Table S1 in Multimedia Appendix 2.

### User Experience

A majority of the sample had a positive attitude toward answering questions about mental health online, with 51% (36/70) scoring 10/10 (very positive; mean 8.41, SD 2.08; range 3-10) and only 4.3% (3/70) scoring in the negative range (ie, <5). Moreover, the method was perceived as easy (mean 8.47, SD 1.98; range 3-10), with 50% (35/70) scoring 10/10 (very easy), whereas 5.7% (4/70) scored it somewhat difficult (ie, <5). Participants scored the questions as easy to understand, with 61.4% (43/70) scoring 5/5 (very easy; mean 4.37, SD 0.90; range 1-5) and only 1 (1.4%) participant answered that it was very difficult (<3). There were no significant differences between language groups.

## Discussion

### Principal Findings

The results from this study show that the i-TAP was very efficient in detecting individuals assessed as having a clinical diagnosis (91.7%) and could correctly identify a total of 82.1% of all positive cases of MDD, anxiety disorder, PTSD, and insomnia disorder, while keeping a low rate of false negatives. The overall accuracy of the i-TAP, ranging between 77.1% and 78.6% for the specific diagnoses and rising to 84.3% for any diagnosis, could be considered commendable, considering the multisymptom screening function and the emphasis on sensitivity. Furthermore, the psychometric performance of the i-TAP is comparable to the performance of the individual instruments included [41,50,58,59], showing that a hierarchical design does not compromise accuracy. In line with previous findings [9,15], our results show a sample with high prevalence of MDD, anxiety disorders, PTSD, and insomnia disorder, as well as a high rate of comorbid disorders. The higher prevalence rates found by the i-TAP compared to the clinical assessment were expected, since the use of self-rating instruments is known to overestimate prevalence [60-62] as they identify symptoms, not disorders. Since the i-TAP is a screening tool, we have prioritized sensitivity over specificity in this validation study. This comes at the cost of a higher number of false positives, which have affected the overall diagnostic test accuracy negatively, and which contribute to the overestimation of prevalence. However, prioritizing specificity would increase the risk of overlooking individuals in need. Weighing sensitivity against specificity for the i-TAP is based on the aim to identify as many individuals as possible, and is built upon the standpoint that, in this case, false positives are less harmful than missing someone in need of mental health services. Against this aim, the i-TAP performed very well. However, the relationship between sensitivity and specificity must be considered for each setting and context, with regard to the purpose and resources for implementation of the i-TAP.

Using a tiered hierarchical model for screening enables the reduction of the number of items an individual responds to, referred to as item burden. Reducing the item burden reduces the time and effort required to complete a screening, thereby increasing the probability of both initiating and completing a screening. Using the i-TAP, an individual with no symptoms would have a reduction in item burden with 69.8% compared to a screening using the full scales, and an individual with, for example, clinically relevant symptoms of anxiety would have 37.2% reduction in item burden. However, for the individuals with comorbid symptoms of all disorders, there is, in fact, an increased item burden, adding the RHS-13 [32] in Tier 1. Despite this, we have weighed the benefits of including the RHS-13 as higher than the costs, in this case, in item burden for individuals with symptoms of all 4 diagnoses. The RHS-13 is a suitable first screener, with culturally sensitive items including somatic symptoms, designed for the general refugee population [31]. In this study, it was shown to be highly sensitive, detecting all but one individual and case of clinical diagnosis, thus serving the purpose of the first tier superbly.

The high prevalence of comorbid diagnoses observed in this sample, as evidenced in several prior studies, for example [11,13], underscores the importance of broad initial screening for refugees. Moreover, the high comorbidity might affect the accuracy of the i-TAP for the specific disorders. Several items and symptoms are similar or shared between instruments and diagnoses, making differential diagnosis difficult in early screening. Regarding anxiety specifically, a positive outcome on the i-TAP can imply any, as well as comorbid, anxiety disorders. Furthermore, the i-TAP falsely identified 8 individuals without any disorder. Hence, the i-TAP cannot be used as a diagnostic tool. Nevertheless, the i-TAP could correctly identify a clear majority of the positive cases for each diagnosis, and all but 3 individuals with a clinical diagnosis. Thus, even if not always precise, the i-TAP can be considered a reliable screening tool for the identification of individuals in need of mental health services.

Regarding the user experience of the i-TAP, results were promising, with a vast majority reporting being positive toward the format and finding the questions easy to complete, indicating acceptability of the i-TAP; however, this needs further investigation.

### Clinical Implications

The results from this study show that the i-TAP could be implemented as a first screener in various clinical settings, such as primary and psychiatric care, schools, health programs, and online mental health services, for individuals with a refugee background in Sweden. In practice, the i-TAP could be useful as a guide to assessment, for symptom-specific interventions, or to tailor support and treatment. For example, combining the i-TAP with a tailored internet-based cognitive behavioral therapy intervention [63], where treatment module provision is based on symptoms, would facilitate automatic adaptation without requiring help-seeking individuals to answer lengthy questionnaires unnecessarily.

In a setting where differentiation of symptoms is less important, the i-TAP could serve as a general screener to identify individuals with any clinically relevant symptoms, and would do so with higher specificity and efficiency compared to using the RHS-13 or full scales only. Applying either approach, the i-TAP’s tiered design could prove especially beneficial in settings with resource and time constraints [25,27]; however, unsuitable in settings where complete data on measures are essential (eg, clinical trials).

Concerning implementation, it is critical to emphasize that the i-TAP is not designed to be a diagnostic instrument and should not be used as such in any context. It is intended as a screening tool, requiring further clinical assessment by a mental health professional in the case of a positive outcome. Moreover, the i-TAP is highly sensitive, and acknowledging the stigma around mental health reported by refugees, we want to highlight the risk of false positive results causing unnecessary distress. Thus, when reporting results back to a patient or participant, it should be done with both cultural and psychometric sensitivity in mind.

Finally, completing the i-TAP requires reading proficiency and digital literacy, and would thus not be a suitable option for all help-seeking individuals with a refugee background; however, adding audio files could be considered to increase applicability. Against this, and the fact that the i-TAP missed 3 individuals with a disorder, we emphasize the need for flexibility and individual adaptation of assessment procedures to promote equity in care.

### Limitations

Some methodological limitations need consideration. The selection of participants was neither randomized nor consecutive, resulting in a convenience sample, which raises questions about bias, representativeness, and generalization. Given the sampling method and self-selection, there is a risk that individuals with mental health problems were more likely to participate, potentially resulting in a higher prevalence compared with the broader population of individuals with a refugee background in Sweden. However, relative to this population, our sample included a higher proportion of asylum-seekers (no residence permit) and individuals residing in asylum facilities—established risk factors for mental ill health [10,11]—which likely contributed to the high prevalence found in this study. The literacy requirement excluded illiterate individuals, who thus are not represented in the sample. Nevertheless, regarding the distribution of sex, age, and countries of origin, the sample was found representative of the Swedish adult population of refugees and asylum-seekers at the time of data collection [29]. While the sample mainly comprised individuals from South West Asia, thereby limiting the generalizability of the findings to these groups, it is likely that the i-TAP could be applicable to other refugee populations currently residing in Sweden, such as Ukrainians, as well as in other contexts. However, this warrants future studies.

Another limitation regards language proficiency as we did not control for literacy. However, the results point to a general understanding of the questions, indicating sufficient language proficiency in the sample. Furthermore, about half of the structured diagnostic interviews were conducted with telephone interpreters, the most common interpretation modality within health care in Sweden [64]. Although clinicians, patients, and interpreters have been found to prefer in-person interpretation for building rapport and trust [65,66], a systematic review found no differences in patient satisfaction between modes of interpretation [67]. Validated translations of the M.I.N.I. would have increased reliability and validity, but were not available. This is the common reality in both research and practice with linguistically diverse groups, and interpreters have assisted M.I.N.I. assessments in previous studies [68,69]. Sharing the Swedish and Arabic translations with interpreters did facilitate the interview, but it was not always possible. Nevertheless, the use of interpreters should not, but always can, influence communication quality and ultimately the clinical assessment [9], and thus pose a limitation in this study.

We did not conduct an interrater reliability check as it was not deemed ethically justifiable to film or ask participants to partake twice, given the vulnerability of the population and study settings [70,71]. It would also pose a risk to recruitment. Measures taken to counter this limitation were: psychologists with previous experience of the M.I.N.I., training, authorized interpreters, assessment conferences, and using structured and validated material. We argue that the range of psychologists and interpreters is a strength in this study, reducing the risk of individual bias in assessments.

Using the M.I.N.I. as the reference standard comes with some limitations. Previous studies show that standardized diagnostic interviews result in more diagnoses when compared to clinical psychiatric assessments [43], thus, the results on prevalence should be interpreted with some caution. Regarding the validation of the i-TAP, we however argue that the sensitivity of the M.I.N.I. [43,44] is suitable, as the objective was to develop and evaluate a sensitive screening tool with the aim of identifying clinically relevant symptoms.

### Conclusion

Our results suggest that the online tiered assessment procedure, i-TAP, can identify clinical depression, anxiety, PTSD, and insomnia efficiently and with high accuracy among individuals with a refugee background residing in Sweden. Implementation of the i-TAP would enable simultaneous screening of the most common psychiatric disorders among refugees and asylum-seekers, reduce the item burden for symptom-free individuals and circumvent decisions on what to screen for. This, together with the digital, multilingual format of the i-TAP, underlines its potential as a feasible, accessible screening tool, with the potential to bridge several of the reported barriers to mental health care.

## Data Availability

The datasets generated or analyzed during this study are not publicly available due to continued analyses and reports but are available from the corresponding author on reasonable request.

## Appendix

Multimedia Appendix 1 Assessment material for insomnia disorder and detailed psychometric evaluation of the Insomnia Severity Index-7.

Multimedia Appendix 2 Extended psychometric evaluation of the internet-based tiered screening procedure.

## Abbreviations

C-SSRS Screen: Columbia Suicide Severity Rating Scale Screening Version
DSM-5: Diagnostic and Statistical Manual of Mental Disorders, Fifth Edition
GAD: generalized anxiety disorder
GAD-2: Generalized Anxiety Disorder-2
GAD-7: Generalized Anxiety Disorder -7
ICD-10: International Statistical Classification of Diseases, Tenth Revision
ISI-7: Insomnia Severity Index
i-TAP: internet-based tiered assessment procedure
MDD: major depressive disorder
M.I.N.I.: Mini International Neuropsychiatric Interview
PCL-5: PTSD (posttraumatic stress disorder) Checklist for DSM-5 (Diagnostic and Statistical Manual of Mental Disorders [Fifth Edition])
PCL-5 SF: PTSD (posttraumatic stress disorder) Checklist for DSM-5 (Diagnostic and Statistical Manual of Mental Disorders [Fifth Edition]) Short Form
PHQ-2: Patient Health Questionnaire-2
PHQ-9: Patient Health Questionnaire-9
PTE: potentially traumatic event
PTSD: posttraumatic stress disorder
RHS-13: Refugee Health Screener -13
RTHC: Refugee Trauma History Checklist
UNHCR: United Nations High Commissioner for Refugees
