# Supplementary material for: Online Tiered Screening for Mental Health Problems Among Refugees in Sweden: Validation Study
Source: JMIR Hum Factors. 2026 Jan 29;13:e82763. doi: 10.2196/82763 (PMC12902762; doi:10.2196/82763)
Supplement: Multimedia Appendix 1 [file humanfactors_v13i1e82763_app1.docx]

**Multimedia Appendix 1. Insomnia**

**Structured clinical interview of insomnia**

*Here are some questions concerning sleep and sleep disturbances*

| **1.** Do you sometimes have trouble falling asleep? | | Yes | No |
| --- | --- | --- | --- |
| **2.** Do you often wake up at night or have problems going back to sleep if you wake up? | | Yes | No |
| **3.** Do you sometimes wake up too early and have difficulty going back to sleep? | | Yes | No |
| If **YES** (1-3): How many times per week do you experience these problems?  If **NO** (1-3): Terminate the interview, circle no symptoms | |  | |
| **4.** Has your sleep quality negatively affected your daily life? For | | Yes | No |
| example, socially, in school or work, activities, or your family life  *(examples of functional impairment: lack of energy, difficulty concentrating, bad mood, difficulty staying awake).*  If **YES**: How much has your sleep quality bothered you?  1-not at all 2-a little 3-quite a lot 4-very much 5-extremely | | | |
| **5.** For how long have you had these sleep difficulties? | | | |
| **6.** What are your conditions for achieving satisfactory sleep? *(For example, is it quiet, dark, or any other factors that are important for sleep)* | | | |
| **Summary of diagnostic criteria**  Yes to 1-3?  Sleep disturbances at least 3 nights/week Yes to functional impairment  Sleep disturbances for at least 3 months Adequate conditions for sleep  Module O in M.I.N.I. summarized Yes/No | **Assessment**  Insomnia disorder Subclinical symptoms  Other specified sleep disorder  No symptoms | | |

# Explorative analysis of cutoffs for ISI 7

For ISI 7, we previously employed a conservative cutoff for detecting

moderate to severe symptoms of insomnia (≥14). However, two more

sensitive cutoffs have been proposed, ≥8 for mild and ≥11 for mild to

moderate insomnia (Morin, 2011, Morin 1999). Aiming to apply the optimal

cutoff, we evaluated the psychometric performance of all three cutoffs with

clinical diagnosis as reference standard, utilising Receiver Operating

Characteristics (ROC). Youden’s *J* and false negatives were used to evaluate

performance of cutoffs, aiming for as high sensitivity and specificity as

possible, with the criteria of keeping sensitivity > specificity.

# Results

ISI 7 showed excellent operating characteristics with area under the curve

(AUC) .859 (95% CI .769-.948). The result of the ROC analysis identified

cutoff ≥14 as having the best overall performance (Youden’s *J* = .564), with

sensitivity 72% and specificity 81.8%, however yielding seven false

negative cases. Cutoff ≥8 showed the lowest overall performance (*J* = .362),

with sensitivity 80.8%, and the lowest specificity of the cutoffs (65.9%),

resulting in five false negatives. Cutoff ≥11 also yielded five false negative

cases and 80.8% sensitivity, specificity was 75.0% and the overall

performance was slightly lower (*J* = .528) compared to cutoff ≥14. Against

these results, our preset criteria, and in alignment with the objective of

identifying clinically relevant symptoms with the i-TAP, cutoff ≥11 for ISI 7

was determined for identification of insomnia in Tier 3 and was thus used

for calculations on the full model. Results of the explorative analysis are

depicted in table 1.

4

29

| **Table S1**. Explorative analysis of ISI-7 in Tier 3 with cutoff 2 for item 7 in Tier 2, calculated with clinical diagnosis according to the structured  clinical interview as reference standard | | | | | | | | | |
| --- | --- | --- | --- | --- | --- | --- | --- | --- | --- |
|  | **npos** | **Kappa** | **Sensitivity**  **(%)** | **Specificity**  **(%)** | **PPV**  **(%)** | **NPV**  **(%)** | **Accuracy**  **(%)** | **Youden’s *J*** | **False negatives** |
| **ISI-7** |  |  |  |  |  |  |  |  |  |
| *Cutoff 8* | 33 | .507 | 80.8 | 72.7 | 63.6 | 86.5 | 75.7 | 36.2 | 5 |
| *Cutoff 11* | 32 | .533 | 80.8 | 75.0 | 65.6 | 86.8 | 77.1 | 52.8 | 5 |
| *Cutoff 14* | 27 | .545 | 72 | 81.8 | 70.4 | 83.7 | 78.6 | 56.4 | 7 |
