# Supplementary material for: Online Tiered Screening for Mental Health Problems Among Refugees in Sweden: Validation Study
Source: JMIR Hum Factors. 2026 Jan 29;13:e82763. doi: 10.2196/82763 (PMC12902762; doi:10.2196/82763)
Supplement: Multimedia Appendix 2 [file humanfactors_v13i1e82763_app2.docx]

| Table S1. Psychometric properties of each tier and disorder in the i-TAP, including accumulative positive and negative cases, with clinical diagnosis according to the structured clinical assessment as reference standard | | | | | | | | | | | | | | | |
| --- | --- | --- | --- | --- | --- | --- | --- | --- | --- | --- | --- | --- | --- | --- | --- |
|  | | **n_pos_** | **kappa** | **Sensitivity (%) (95% CI)** | **Specificity (%)  (95% CI)** | **PPV (%)  (95% CI)** | **NPV (%)  (95% CI)** | **Accuracy (%)** | **TP** | | **TN** | | **FN** | | **FP** |
| **TIER 1 Identification (cutoff)** | | | | | | | | | | | | | | | |
| RHS-13 (11) | | 50 |  | | | | | | | | | | | | |
| *Any diagnosis** |  | .537 | 97.2 | 55.9 | 70.0 | 95.0 | 77.1 | 35 | 19 | | 1 | | 15 | |  |
| *Depression* |  | .276 | 100 | 40.0 | 40.0 | 100 | 57.1 | 20 | 20 | | 0 | | 30 | |  |
| *Anxiety* |  | .181 | 94.1 | 35.8 | 32.0 | 95.0 | 50.0 | 16 | 19 | | 1 | | 34 | |  |
| *PTSD* |  | .276 | 100 | 40.0 | 40.0 | 100 | 57.1 | 20 | 20 | | 0 | | 30 | |  |
| *Insomnia* |  | .382 | 100 | 45.5 | 52.0 | 100 | 65.7 | 26 | 20 | | 0 | | 24 | |  |
| **TIER 2 Symptom differentiation (cutoff)** | | | | | | | | | | | | | | | |
| *Any diagnosis** | 49 | .566 | 97.2 | 58.8 | 71.4 | 95.2 | 78.6 | 35 | 20 | | 1 | | 14 | |  |
| *Depression* PHQ-2 (2) | 43 | .401 | 100 | 54.0 | 46.5 | 100 | 67.1 | 20 | 27 | | 0 | | 23 | |  |
| *Anxiety* GAD-2 (2) | 34 | .360 | 92.9 | 62.5 | 38.2 | 97.2 | 68.6 | 13 | 35 | | 1 | | 21 | |  |
| *PTSD* PCL 5 SF (5) | 31 | .549 | 90.0 | 74.0 | 58.1 | 94.9 | 78.6 | 18 | 37 | | 2 | | 13 | |  |
| *Insomnia* ISI item 7 (2) | 34 | .539 | 84.6 | 72.7 | 64.7 | 88.9 | 77.1 | 22 | 32 | | 4 | | 12 | |  |
| **TIER 3 Severity indication (cutoff)** | | | | | | | | | | | | | | | |
| *Any diagnosis** | 41 | .684 | 91.7 | 76.5 | 80.5 | 89.7 | 84.3 | 33 | 26 | | 3 | | 8 | |  |
| *Depression PHQ-9 (10)* | 33 | .561 | 95.0 | 72.0 | 57.6 | 97.3 | 78.6 | 19 | 36 | | 1 | | 14 | |  |
| *Anxiety GAD-7 (10)* | 25 | .464 | 76.5 | 77.4 | 52.0 | 91.1 | 77.1 | 13 | 41 | | 4 | | 12 | |  |
| *PTSD PCL 5 (32)* | 27 | .525 | 80.0 | 78.0 | 59.3 | 90.7 | 78.6 | 16 | 39 | | 4 | | 11 | |  |
| *Insomnia ISI 7 (11)* | 32 | .533 | 80.8 | 75.0 | 65.6 | 86.8 | 77.1 | 21 | 33 | | 5 | | 11 | |  |
| **Positive case for any diagnose  n_pos_* positive cases for each measure and cases forwarded to the next tier, *CI* confidence interval*, PPV* positive predictive value*, NPV* negative predictive value*, Accuracy* proportion of true positives and true negatives, *TP* true positive*, TN* true negative*, FN* false negative*, FP* false positive. | | | | | | | | | | | | | | | |

**Multimedia Appendix 2. Extended psychometric evaluation of the i-TAP**
